# Myelotoxicity of Temozolomide Treatment in Patients with Glioblastoma Is It Time for a More Mechanistic Approach?

**DOI:** 10.3390/cancers15051561

**Published:** 2023-03-02

**Authors:** Medhat M. Said, Martinus P. G. Broen, Eleonora L. Swart, Imke H. Bartelink, Mathilde C. M. Kouwenhoven

**Affiliations:** 1 Department of Pharmacy & Clinical Pharmacology, Amsterdam UMC Location Vrije Universiteit Amsterdam, De Boelelaan 1117, 1081 HV Amsterdam, The Netherlands; 2 Cancer Treatment and Quality of Life, Cancer Center Amsterdam, De Boelelaan 1118, 1081 HV Amsterdam, The Netherlands; 3 Department of Neurology, Maastricht University Medical Center, 6202 AZ Maastricht, The Netherlands; 4 GROW-School for Oncology and Reproduction, Maastricht University, 6229 ER Maastricht, The Netherlands; 5 Department of Neurology, Amsterdam UMC Location Vrije Universiteit Amsterdam, 1081 HV Amsterdam, The Netherlands

## 1. Introduction

**Figure 1:**
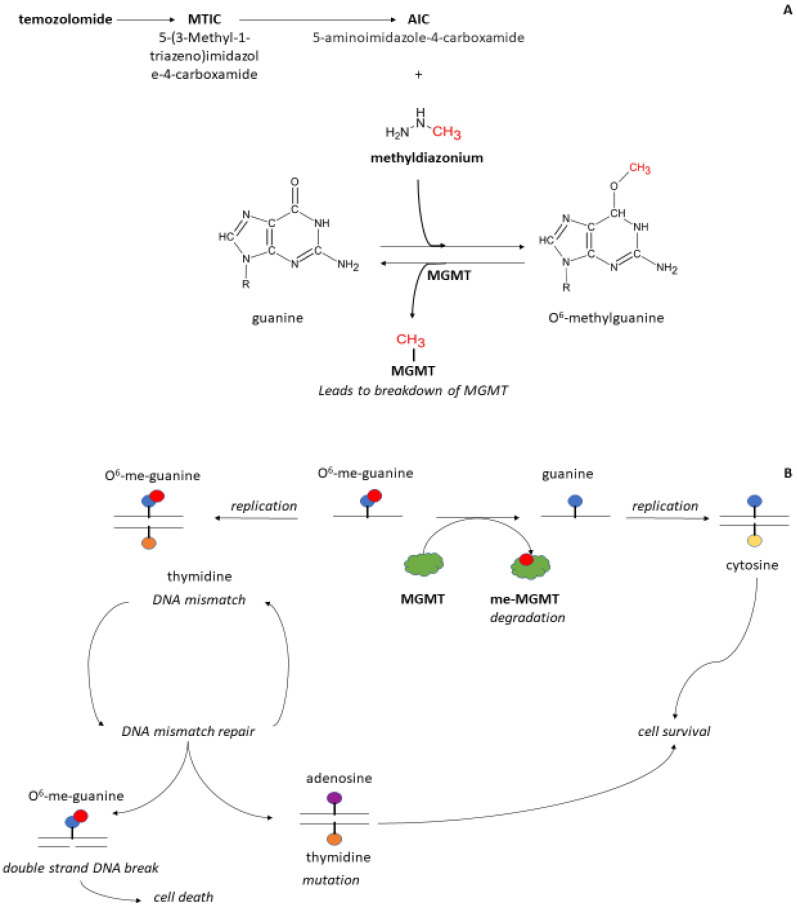
Schematic of the mechanism of action of temozolomide (panel **A**) and O^6^-methyl-guanyl methyltransferase (panel **B**) on DNA. Temozolomide (panel **A**) is broken down under acid conditions (pH < 4) into 5-(3-Methyl-1-triazeno)imidazole-4-carboxamide (MITC). When pH raises in the gut (pH > 6) MITC is further converted into 5-aminoimidazole-4-carboxamide (AIC) and methyldiazonium. Methyldiazonum donates a methyl group to the O^6^-position of guanine resulting in DNA methylation. The enzyme methyl-guanyl methyl transferase (MGMT, panel **B**) is a protein that can remove the added methyl group on guanine. Thereafter, MGMT is broken down and inactivated. When replication occurs, guanine pairs with cytosine. Upon methylation, O^6^-guanine is paired with thymidine. This interaction leads to a mismatch signal and activation of mismatch repair enzymes. If the O^6^-methylated guanine is thoroughly removed and the DNA repaired, guanine can pair with cytosine without DNA damage. When O^6^-guanine is not repaired and thymidine is paired to this group, a mutation occurs through replacement by adenosine. When no bases are paired with the O^6^-guanine, the result is a break in the DNA that eventually leads to cell death.

Glioblastoma multiforme is the most common primary central nervous system tumor, with an incidence of 3.2 per 100,000 per year [1]. Patients with glioblastoma have a poor two-year survival rate of approximately 17% [2]. Resection is usually the first step in treating patients with glioblastoma. After the surgery, patients receive six weeks of radiotherapy and concurrent temozolomide chemotherapy. The final step in the treatment protocol consists of six adjuvant temozolomide courses. Temozolomide is an alkylating agent that functions as a prodrug. Upon ingestion, temozolomide breaks into its active intermediary, 5-(3-methyl-1-triazeno) imidazole-4-carboxamide (MTIC). MTIC causes DNA methylation at the O^6^ position on guanine in the DNA and eventually leads to cell death. The naturally occurring enzyme O^6^-methylguanine-DNA methyltransferase (MGMT) can repair the MTIC-inflicted DNA methylation, resulting in cell survival (Figure 1).

Approximately 50% of patients with glioblastoma have tumors with a methylated MGMT promotor. These patients have less active MGMT and benefit the most from temozolomide treatment. In contrast, patients with an unmethylated MGMT promoter do not benefit much from adding temozolomide [3].

After disease progression, myelotoxicity is the most frequent cause of treatment adjustment and abrogation [2]. Idiosyncratic drug reactions such as aplastic anemia and treatment-induced myelodysplasia also occur in temozolomide-treated patients. These rare reactions have a high fatality rate (up to 60%) due to an increased risk of internal bleeding and septicemia [4]. At present, it is unclear which patients will develop severe myelotoxicity during temozolomide treatment and subsequently need toxicity-dictated adjustments to the treatment. A more systematic approach towards identification of predictors of myelotoxicity may allow timely treatment modifications, reduce the severity of myelotoxicity, and prevent treatment delays and abrogation.

## 2. Myelotoxicity Is Not a Rare Event and Leads to Significant Treatment Alterations

In a recent review of multiple clinical trials, including the original milestone study [2], most patients experienced some form of myelotoxicity after temozolomide treatment [5]. Lymphopenia was most common and occurred in 81.2%, followed by anemia in 44.7% of patients. Thrombocytopenia and neutropenia were observed in 26.5% and 18.9% of patients, respectively. Severe myelotoxicity is defined as a grade 3 or higher in the Common Terminology Criteria for adverse events (CTCAE) [6] and occurred in a considerable proportion (16–23%) of patients [2,7,8,9,10]. Subjects enrolled in these clinical trials do not necessarily represent the patients in clinical practice. For example, in an extensive population-based Norwegian survey, only 43% of patients with newly diagnosed glioblastoma fulfilled the original trial criteria [11].

Several studies describe the occurrence of myelotoxicity in practice-based populations. The most extensive retrospective cohorts comprised 680 [12] and 300 [13] patients treated with temozolomide. In these studies, 6–11% of patients developed severe myelotoxicity. This proportion is close to that reported in the original trial [2]. In contrast to the initial trials, most myelotoxicity occurred during or shortly after the concomitant treatment phase [14,15]. Half of the patients with severe thrombocytopenia are at risk of sustained, prolonged, and potentially irreversible toxicity [14]. Up to 54% of these patients need blood or platelet transfusions or growth factor treatment [14,15,16]. In addition, patients with severe toxicity in one cell subset frequently have deficiencies occur in multiple cell subsets simultaneously [16,17]. Moreover, most patients (57–90%) have treatment interruptions [14,18] and approximately 23% (95% CI, 13%–37%) [12,14,15] of patients stop treatment due to myelotoxicity [11,14]. These studies show that severe myelotoxicity is a common event during the treatment of patients with glioblastoma, that may impact treatment burden of patients. However, these studies also included significant numbers of patients who received prior chemotherapy [12]. Inclusion of such heterogeneous patient populations should be considered with care as patients who develop severe myelotoxicity during first-line treatment receive second-line chemotherapy, infrequently leading to potential selection bias [14,18].

## 3. A More Systematic Approach towards Myeloxicity

Temozolomide, like most chemotherapeutics, is dosed based on Body Surface Area (BSA). The mechanisms underlying myelotoxicity are still poorly defined and focus generally on identification of risk factors from retrospective patient cohorts or as part of clinical trials. In most clinical trials, women have a much higher risk of developing temozolomide-induced myelotoxicity than men [4,5,12,14,15,16,19,20]. These studies indicate that the risk of temozolomide-induced myelotoxicity is modified differentially in men and women and might benefit from a stratified analysis. Armstrong [12] performed such analysis and observed that men with a BSA ≥ 2 m^2^ and age > 40 years had a higher risk on myelotoxicity. Meanwhile, for women, BSA < 2 m^2^ and age 31–40 years were independent risk factors [12]. Other factors associated with increased risk of myelotoxicity included: serum creatinine [16], baseline leukocyte, and platelet [3,21] and absolute lymphocyte counts [3]. Although these factors have been identified, they do not explain the mechanisms underlying myelotoxicity. Considering the heterogeneity of drug response among patients, a better understanding of the exposure–toxicity relationship is necessary.

**Figure 2:**
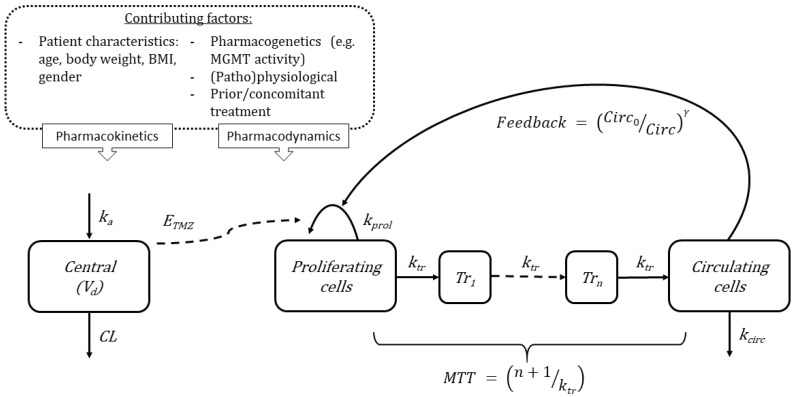
Schematic of proposed mathematical model for myelosuppression after TMZ treatment. This pharmacokinetics–pharmacodynamics model can be expanded to describe the reduction of different cell lineages such as platelets, red blood cells, and white blood cells. Variables: k_a,_ absorption constant rate; V_d_, apparent distribution volume; CL, apparent clearance; E_TMZ_, Temozolomide drug effect; k_tr_, maturation rate constant; k_prol_, proliferation rate constant; Tr_1_, transition compartment 1; Tr_n_, transition compartment; k_circ_, degradation rate constant; Circ_0_, circulating blood cells at baseline; Circ, amount of circulating blood cells; MTT, mean transit time; and γ as the inverse feedback loop parameter. The model consists of a proliferating compartment that is sensitive to TMZ, transit compartments represent maturation, and a compartment of circulating blood cells. The contributing covariates known to affect TMZ treatment.

The observed differences are likely based on differences in the pharmacokinetics (PK) and pharmacodynamics (PD) of anticancer therapies. Temozolomide has a short half-life (2 h) with predictable linear pharmacokinetics. Gender and BSA independently influence the clearance of oral temozolomide. Men have a faster temozolomide clearance than women, and patients with a higher BSA have a higher clearance than those with lower BSA [22,23]. Including variables such as age and BSA in a validated PK-myelotoxicity model for temozolomide provides a more mechanistic approach towards myelotoxicity, in which both patient characteristics and drug properties are considered simultaneously. Friberg [24] developed the PK-PD model to describe chemotherapy-induced myelosuppression. This semi-mechanistic model has been frequently used in the development of anticancer drugs [21] and specifically for temozolomide [25] (Figure 2). Using a similar PK-PD model, Panetta et al., found that, rather than just blocking stem cell production, temozolomide was cytotoxic in the bone marrow [25]. This mechanism is further supported by studies that show that non-proliferative blood cells were not susceptible to the damage of temozolomide [26]. Using these models, the area under the curve (AUC) of temozolomide appeared to explain the reduction, the nadir, and the rebound effect of absolute neutrophil count after temozolomide dosing in children < 16 years old [25]. Such models can be expanded to adult populations and when combined with extensive covariate analyses, may improve our understanding of contribution factors of PK and PD (Figure 2 top). When appropriately validated, models can be used to predict the additive myelotoxic effect of new drug combinations, such as the promising combination treatment of temozolomide and veliparib [27]. Furthermore, the association between toxicity and efficacy can be included in these analyses. Models as described above could not only aid decision-making on dosing regimens to decrease treatment interruptions due to severe myelotoxicity, but could also expand the treatment options for severe myelotoxicity that are currently limited to dose delays, reductions of temozolomide, and administration of growth factors [28,29].

## 4. Should MGMT Be Included in the PK-PD Model for Myeloxicity?

Preclinical studies show that MGMT-deficient glioblastoma cell lines have significantly enhanced cytotoxicity during concurrent radiation and temozolomide compared to MGMT-proficient cells [30]. When MGMT is depleted, cells become more sensitive to the toxic effects of temozolomide. Upon restoring MGMT function through transfection with cDNA, this cytotoxic effect is reversed [31]. These data are confirmed in daily practice as patients with low MGMT activity within their tumors have improved prognoses [3].

There is high variability in MGMT activity among and within different tissues; the highest activity levels are observed in the liver, and low levels in the brain and bone marrow are precursors [31,32]. MGMT activity within peripheral blood mononuclear cells (PBMC) is considered a good surrogate for MGMT activity in bone marrow-residing progenitor cells [31]. MGMT activity in PBMCs in a healthy population shows a considerable inter-individual variation, but only a moderate intra-individual variation over several weeks [33]. The differential MGMT activity among individuals may rely on recently identified single nucleotide polymorphisms (SNPs) in the MGMT gene that modify the enzymatic activity of MGMT [34,35,36].

Clinical studies showed that MGMT activity in PBMCs can decline with more than 50% during temozolomide treatment and patients with lower pretreatment MGMT expression in PBMC more often experienced severe myelotoxicity [19,37,38,39,40]. In particular, patients that carry certain SNPs within the MGMT gene, of which some were linked to lower MGMT activity, had an increased risk of myelotoxicity when treated with temozolomide [12,41]. Patients carrying multiple risk alleles of MGMT had an increased risk of myelotoxicity of up to 240% [41]. Taken together, these data suggest that inclusion of MGMT activity in PBMCs in a PK-PD-model is attractive and may serve as promising a biomarker to predict and monitor the individualized risk of myelotoxicity.

## 5. Conclusions

Individualized comprehensive PK-PD-based models can aid identification of patients at risk for severe myelotoxicity and give clinicians tools for individualized dosing regimens to reduce the occurrence of severe myelotoxicity, while maintaining temozolomide efficacy in selective patient groups. PK-PD models use both knowledge of temozolomide’s pharmacokinetics and dynamics and patients’ characteristics and may provide a more mechanistic base for understanding temozolomide toxicity. MGMT activity in PBMCs forms an promising biomarker for prediction and early detection of myelotoxicity, and its relation to myelotoxicity should be defined.
